# Structure-Based Prediction of Molecular Interactions for Stabilizing Volatile Drugs

**DOI:** 10.3390/pharmaceutics18010111

**Published:** 2026-01-15

**Authors:** Yuchen Zhao, Danmei Bai, Boyang Yang, Tiannuo Wu, Guangsheng Wu, Tiantian Ye, Shujun Wang

**Affiliations:** 1 Department of Pharmaceutics, School of Pharmacy, Shenyang Pharmaceutical University, Shenyang 110016, China; zhaoyuchen202110@163.com (Y.Z.); 17678391667@163.com (D.B.); yby_2003@163.com (B.Y.); guangsheng12138@163.com (G.W.); 2 School of Medicine, Guangxi University, Nanning 530004, China; 13940536545@163.com

**Keywords:** structure prediction model, molecular interaction prediction, high-throughput screening of excipients, liposome, volatile drug stabilization

## Abstract

**Background/Objectives**: The high volatility of volatile drugs significantly restricts their clinical applicability. Although excipients capable of strong interactions can reduce volatilization, conventional screening methods rely on empirical trial-and-error, resulting in low efficiency and high resource consumption. To address this limitation, this study introduces an artificial intelligence (AI)-driven strategy for screening drug–excipient interactions. Using d-borneol as a model drug, this approach aims to efficiently identify strongly interacting excipients and develop stable nano-formulations. **Methods**: High-throughput simulations were performed using the Protenix structure prediction model to evaluate interactions between d-borneol and 472 FDA-approved excipients. The top 50 candidate excipients were selected based on these simu-lations. Molecular docking and stability experiments were conducted to validate the predictions. **Results**: Molecular docking and stability experiments confirmed the consistency between predicted and experimental results, validating the model’s reliability. Among the candidates, soybean phospholipid (PC) was identified as the optimal excipient. A lyophilized liposomal formulation prepared with PC significantly suppressed the volatilization of d-borneol and improved both thermal and storage stability. Mechanistic investigations indicated that d-borneol stably incorporates into the hydro-phobic region of phospholipids, enhancing membrane ordering via hydrophobic interactions without disturbing the polar headgroups. **Conclusions**: This study represents the first application of a structure prediction model to excipient screening for volatile drugs. It successfully addresses the stability challenges associated with d-borneol and offers a new paradigm for developing nano-formulations for volatile pharmaceuticals.

## 1. Introduction

Volatile drugs (VDs), primarily monoterpenes, sesquiterpenes, and their derivatives, have garnered considerable interest owing to their notable biological activities—including anti-inflammatory, antibacterial, antioxidant, and analgesic effects [1]. However, their high volatility and susceptibility to chemical degradation lead to substantial losses during production and storage, compromising the efficacy of the final products [2,3]. Furthermore, their hydrophobic nature contributes to low bioavailability, which further restricts their clinical applicability [4]. Consequently, achieving adequate stability and concentration of these active compounds while enhancing their water solubility and bioavailability is essential for improving therapeutic outcomes, representing a crucial unmet clinical need [5].

Currently, the primary approach for stabilizing VDs involves cyclodextrin (CD) inclusion complexes [6,7]. However, the clinical translation of this strategy is limited by inherent drawbacks, such as saturation effects in host–guest interactions and limited adaptability to complex formulations [8]. Nanocarriers have been widely explored as delivery systems for VDs to enhance their stability and bioavailability [9,10,11,12,13,14,15]. Nonetheless, the development process remains largely empirical, relying on trial-and-error screening of excipient combinations, which is time-consuming, resource-intensive, and inefficient [16,17,18]. Although conventional computational approaches, such as molecular docking, have improved screening efficiency, they are constrained by limited force-field accuracy and inadequate modeling of multi-ligand synergy [19,20,21,22]. Moreover, no previous study has employed artificial intelligence (AI)-based structure prediction models—particularly diffusion models used in protein–ligand interaction prediction—to investigate drug–excipient interactions. However, the high-precision modeling of non-covalent interactions—such as hydrogen bonding and hydrophobic effects—which these models excel at, is precisely the key to rationally predicting the compatibility and binding stability between drugs and excipients.

**Figure 1:**
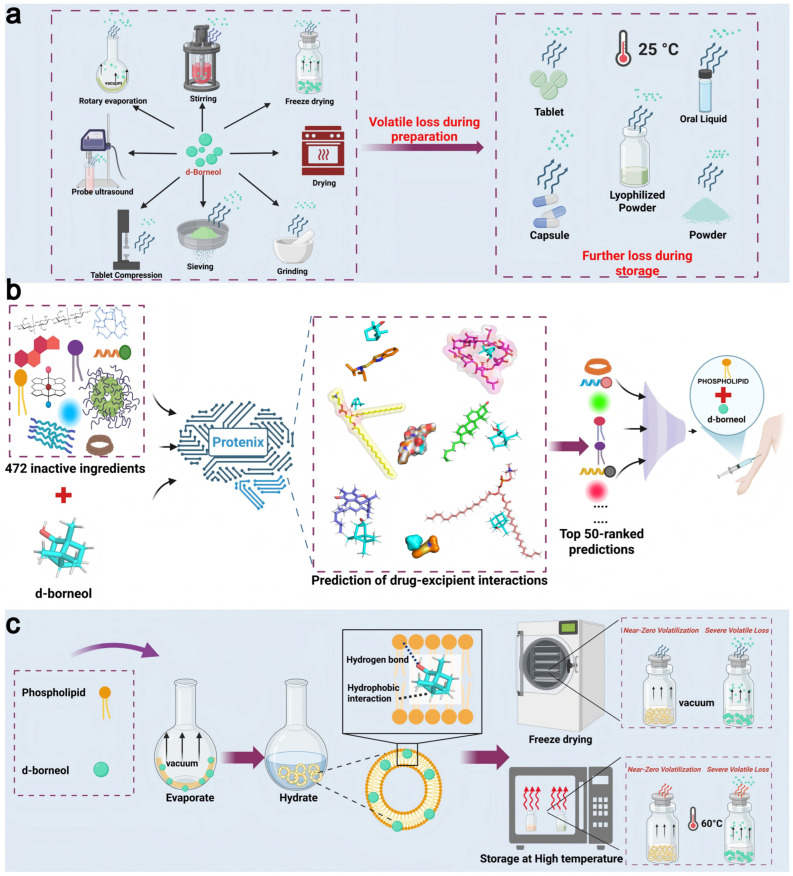
Schematic of the AI-driven excipient screening and formulation design strategy for inhibiting d-borneol volatilization. (**a**) Volatilization pathways of d-borneol during processing (e.g., rotary evaporation, freeze-drying) and storage (e.g., in tablets or lyophilized powders). (**b**) High-throughput screening of excipients using the Protenix model: ligand–ligand interactions between d-borneol and 472 FDA-approved excipients were simulated to identify the top 50 candidates. (**c**) Phospholipids interact with d-borneol hydrophobically through their acyl chains. At higher concentrations, they self-assemble into bilayers that encapsulate d-borneol, thereby significantly suppressing its volatility.

AlphaFold (developed by Google DeepMind) achieves high-accuracy prediction of protein three-dimensional structures from amino acid sequences alone by integrating attention mechanisms with evolutionary sequence analysis. Its prediction accuracy in the CASP competition has approached experimental levels, marking a revolutionary shift in the field of computational biology [23]. The next-generation model, AlphaFold3, extends its applicability to diverse biomolecular systems—including proteins, nucleic acids, and small molecule ligands—and further enhances prediction accuracy by incorporating a diffusion model. Although this series of models demonstrates the strong potential of deep learning in elucidating molecular interactions, their design is not specifically optimized for large-scale, industrial virtual screening scenarios. In particular, AlphaFold3 cannot meet the core requirements of this study for rapid, batch computation of massive pharmaceutical excipients, as well as flexible and stable local deployment [24]. Therefore, this study employs the Protenix model, developed by ByteDance, to perform high-throughput simulations. Protenix inherits the core diffusion architecture and Evoformer module of AlphaFold3, while owing to its fully open-source nature and targeted optimizations, significantly reduces computational overhead and reliably supports efficient local deployment and large-scale concurrent processing [25,26]. These characteristics make Protenix particularly suitable for large-scale virtual screening of pharmaceutical excipients, enabling direct prediction of the interaction patterns and strengths between drug molecules and excipients, thereby serving as an ideal computational tool for this study.

d-Borneol, a representative VD [27], possesses diverse biological activities and can facilitate blood–brain barrier penetration of co-administered drugs [28,29,30], highlighting its value in combination therapies. Nevertheless, its high volatility results in substantial loss during manufacture and storage [31]. In addition, its poor water solubility and low bioavailability further hinder clinical application [32,33]. In this study, we implemented an AI-driven strategy for predicting molecular interactions, using d-borneol as a model drug. We applied the Protenix model to conduct high-throughput simulations of interactions between d-borneol and 472 FDA-approved excipients. Excipients exhibiting strong interactions were identified, and subsequent thermal stability and freeze-drying studies led to the selection of soybean phospholipid as an optimal nanocarrier excipient. An injectable d-borneol liposomal system was developed, leveraging strong drug–phospholipid interactions and bilayer encapsulation to significantly suppress volatility and improve bioavailability(Figure 1). This approach offers a new paradigm for formulating stable VD-based therapeutics.

## 2. Materials and Methods

### 2.1. Materials

d-Borneol (purity 99.5%) was donated by Jiangsu Kanion Pharmaceutical Co., Ltd. (Lianyungang, China) Soybean phospholipids S100 (SPC, purity ≥ 94%) were purchased from Lipoid GmbH (Ludwigshafen, Germany). Sodiumdeoxycholate (SDC), N,N-diisopropyl-2-benzothiazolesulfenamide (DBTS), and levulinic acid (La) were purchased from Bide Pharmatech (Shanghai, China). Sucrose was purchased from Hunan Erkang Pharmaceutical (Changsha, China). Hydroxypropyl-β-cyclodextrin was purchased from Shanghai LuFu Pharmaceutical Technology Co., Ltd. (Shanghai, China). All organic solvents used were of analytical grade.

### 2.2. Excipient Database Screening

The initial FDA Excipient Database contained 9303 entries. (https://www.fda.gov/drugs/drug-approvals-and-databases/inactive-ingredients-database-download, accessed on 14 January 2025). To prepare a structured molecular library for high-throughput simulation, a series of custom Python scripts was developed to automate the data curation pipeline. First, the ingredient names and their corresponding Unique Ingredient Identifiers (UNIIs) were extracted from the raw data file. A script was then executed to remove duplicate entries based on the UNII field, ensuring the uniqueness of each chemical entity. Subsequently, a second script was employed to programmatically merge this deduplicated list with a separate reference database containing standardized two-dimensional structural information (SMILES strings). This matching was performed via the UNII key to associate each excipient with its canonical molecular representation. Following this association, a final filtering script applied two key criteria: (1) removal of all entries lacking a valid SMILES string, and (2) exclusion of records whose SMILES string consisted solely of a single sulfur (S) element, as these were deemed irrelevant for typical molecular interaction studies. Through this automated and standardized data processing workflow, a final set of 472 FDA-approved excipients with well-defined chemical structures was obtained and retained for subsequent AI-driven interaction prediction.

### 2.3. Prediction and Screening of d-Borneol-Excipient Interactions Based on Protenix

All simulations were performed in a local Linux environment (Ubuntu 24.04.1 LTS) using custom Python scripts (v3.10.16) to interface with ByteDance’s Protenix (https://protenix-server.com/login, accessed on 15 January 2025) platform for large-scale drug–excipient interaction prediction. The molecular type was specified as ligand. SMILES strings of d-borneol and each of the 472 excipients were input, and interactions were simulated at a 1:1 molar ratio. The excipients were ranked based primarily on the interface predicted TM score (ipTM), and secondarily on the ranking_score. The top 50 candidates were selected. Subsequently, the molar ratio of d-borneol to excipient was changed to 1:5, and these 50 excipients were re-evaluated and re-ranked, yielding the final list of top 50 excipients. (The complete code files are available in the Supplementary Materials).

### 2.4. Validation of Excipient-d-Borneol Interaction Prediction Based on Molecular Docking

Molecular docking validation was performed using AutoDock Vina 1.2.6. In this setup, d-borneol was defined as the receptor, and the 50 selected excipients served as ligands. All ligands and the receptor underwent preprocessing, including hydrogen addition, charge assignment, and energy minimization. A docking grid box was configured to fully encompass the d-borneol molecule, ensuring adequate sampling space. AutoDock Vina was then executed to compute the predicted binding affinity (in kcal/mol) for each excipient–d-borneol complex. To assess the consistency between the two computational methods, Spearman correlation analysis was applied to evaluate the statistical relationship between the predicted binding affinities and both the ipTM values and ranking scores obtained from Protenix.

### 2.5. Experimental Evaluation of the Physical Stability of d-Borneol–Excipient Complexes

Based on the screening results, five candidate excipients were selected from the final top 50 list: hydroxypropyl-β-cyclodextrin (HP-β-CD, ranked 2nd), sodium deoxycholate (SDC, ranked 16th), soybean lecithin (PC, ranked 33rd), N,N-diisopropyl-2-benzothiazolesulfenamide (DBTS, ranked 50th), and—as a control—levulinic acid (La, ranked 471st). The complexes were prepared using low-temperature reduced-pressure rotary evaporation. d-Borneol and each excipient were dissolved in anhydrous ethanol at 1:1 and 1:5 molar ratios. The organic solvent was then removed using a rotary evaporator equipped with a 30 °C water bath, resulting in the formation of solid thin films.

#### 2.5.1. Freeze-Drying Retention Rate

Equal amounts of each complex were subjected to freeze-drying. The d-borneol content was measured by gas chromatography (GC) before and after freeze-drying to calculate the low-temperature solid-state retention rate.

#### 2.5.2. Thermal Acceleration Loss Rate

For thermal stability assessment, the complexes were sealed in glass vials and heated at 60 °C for 12 h in a constant-temperature oven. The residual amount of d-borneol before and after heating was quantified using GC to evaluate its volatility under high-temperature conditions.

### 2.6. Sample Preparation

Small unilamellar liposomes of soybean phospholipids S100 and d-borneol/soybean phospholipids S100 at different molar ratios were prepared by the thin-film hydration-ultrasonication method. Appropriate amounts of soybean phospholipids S100 and d-borneol were weighed and dissolved in ethanol to prepare stock solutions of soybean phospholipids S100 and d-borneol. Stock solutions of soybean phospholipids S100 and d-borneol were pipetted into a 100 mL round-bottom flask at different molar ratios; a thin film was formed by rotary evaporation under vacuum drying. Then, 10 mL of 0.3% (*W*/*V*) sucrose solution was added, and the mixture was stirred in a 50 °C water bath for 30 min, followed by ultrasonication with an ultrasonic cell disruptor (300 W) for 10 min to form small unilamellar liposomes with uniform particle size.

### 2.7. Molecular Dynamics Simulation of Bo-Lip at Different Molar Ratios

All molecular dynamics simulations were performed using the Gromacs 2022 software package. The initial structure of the system was constructed using Packmol v20.16.1, containing d-borneol and POPC phospholipid bilayers at different molar ratios (1:1, 1:5, 1:10, 1:15), with the TIP3P water model as the solvent and an ion concentration of 150 mM NaCl. The CHARMM36 force field was adopted, and parameters for d-borneol were generated by CGenFF. The simulation process included: energy minimization (steepest descent and conjugate gradient methods); NVT and NPT equilibration; and finally, 100 ns of unrestrained production simulation (time step of 2 fs, LINCS algorithm for hydrogen bond constraint, semi-isotropic pressure coupling). Each system was simulated independently three times.

### 2.8. Characterization of d-Borneol Liposomes (Bo-Lip) at Different Molar Ratios

The particle size distribution of d-borneol liposomes at different molar ratios was measured using a Malvern Zetasizer Nano ZS90 (Malvern Instruments Ltd., Malvern, UK). The liposomes were stained with 2% phosphotungstic acid, and then a transmission electron microscope (TEM) (HITACHI 7700; Hitachi High-Technologies Corporation, Tokyo, Japan) was used to observe and image the morphology of the liposomes.

### 2.9. Thermodynamic Characterization by Differential Scanning Calorimetry

A TA DSC2500 differential scanning calorimeter (TA Instruments, New Castle, DE, USA) was used for DSC analysis, with a temperature accuracy of ±0.025 °C and an enthalpy accuracy of ±0.04%. The atmosphere was nitrogen, and the scanning rate was 1 °C/min. Measurements were repeated at least three times to ensure reproducibility.

### 2.10. Molecular Interaction Analysis by Fourier Transform Infrared Spectroscopy

A Thermo Fisher Scientific Nicolet iS20 (Waltham, MA, USA) was used for FTIR analysis. In this experiment, the wavenumber range was 400–4000 cm^−1^, the resolution was 4 cm^−1^, and each spectrum was scanned 32 times. Liposome samples with different molar ratios of d-borneol were uniformly coated on the surface of KBr salt plates, which were then placed on the sample holder for the experiment.

### 2.11. Thermal Stability Evaluation by Thermogravimetric Analysis

A HITACHI STA200 thermogravimetric analyzer (Tokyo, Japan) was used under a nitrogen atmosphere with a flow rate of 100 mL/min. Samples weighing 10 mg ± 0.5 mg were placed in an open alumina crucible and heated from 30 °C to 300 °C at a heating rate of 5 °C/min. TGA/DTA curves were obtained.

### 2.12. Data Analysis

Experimental results were expressed as mean ± standard deviation (mean ± SD). Differences between groups were analyzed using Python (version 3.10), SciPy library (version 1.11), and Pandas library (version 2.1): raw data were first organized using the Pandas library, and then Levene’s test was performed using the SciPy library to verify variance homogeneity. For homogeneous variances, one-way analysis of variance (one-way ANOVA) combined with Tukey’s HSD test was used for post hoc analysis. Statistical significance was set as follows: * *p* < 0.05, ** *p* < 0.01, *** *p* < 0.001, **** *p* < 0.0001 (ns: no significance).

## 3. Results and Discussion

### 3.1. Prediction and Screening of d-Borneol-Excipient Interactions Based on Molecular Interaction Principles

**Figure 2:**
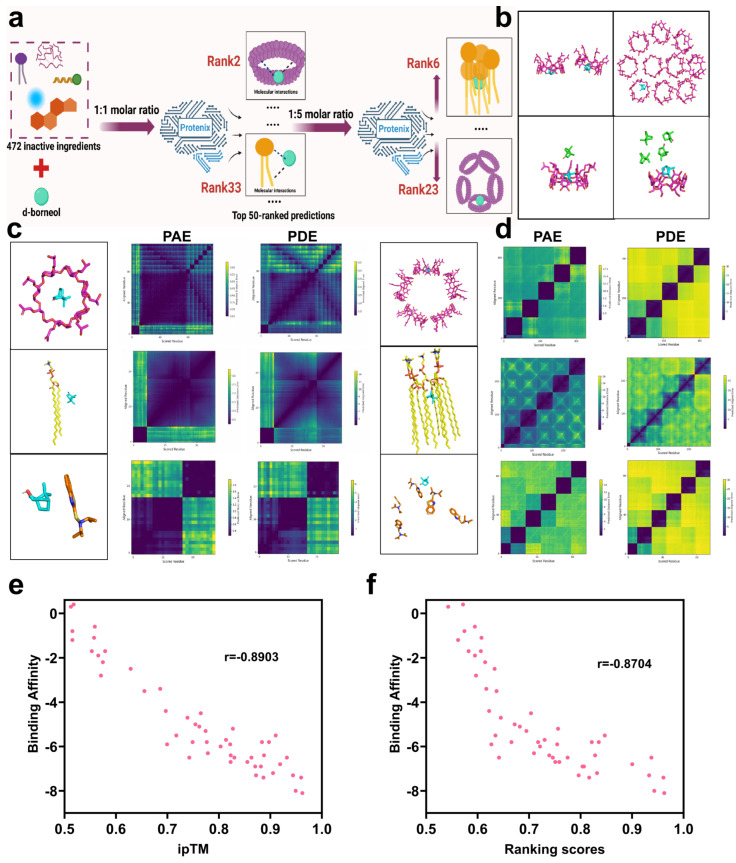
Simulation analysis of intermolecular interactions between d-borneol and selected excipients. (**a**) Schematic of the excipient screening process. (**b**) Three-dimensional representation of d-borneol and hydroxypropyl-β-cyclodextrin at varying molar ratios. (**c**) Predicted distance error (top) and alignment error (bottom) heatmaps for d-borneol with hydroxypropyl-β-cyclodextrin, phospholipid, and N,N-diisopropyl-2-benzothiazolesulfenamide at a 1:1 molar ratio. (**d**) Predicted distance error (top) and alignment error (bottom) heatmaps for the same excipients with d-borneol at a 1:5 molar ratio. (**e**) Correlation of ipTM with binding affinity. (**f**) Correlation of ranking score with binding affinity.

To identify excipients capable of optimizing the formulation performance of d-borneol (API), this study employed the Protenix model to predict and analyze drug–excipient interactions based on interface binding characteristics. The interface predicted TM score (ipTM), which accurately estimates inter-chain interface accuracy [34], was designated as the primary evaluation criterion. The ranking_score—a composite metric integrating ipTM, pTM, and pLDDT to reflect overall prediction reliability [35]—served as the secondary criterion. Using these criteria, the predicted structural quality of 472 excipients was comprehensively evaluated and ranked in descending order, leading to the selection of the top 50 excipients with strong binding affinity (Table S1) (Figure 2a). In practical formulations, excipients are required in larger quantities than the API to serve roles such as stabilizers and release modifiers. Since the Protenix model at a 1:1 molar ratio primarily reflects molecular-level binding characteristics, it predicts static binding potential. To better approximate real formulation conditions and enhance predictive accuracy, the top 50 excipients were re-evaluated at a 1:5 molar ratio, followed by re-ranking based on interaction strength (Table S2).

To validate the prediction accuracy of the Protenix model, three representative excipients—covering a range of rankings—were selected: hydroxypropyl-β-cyclodextrin (Hp-β-CD, ranked 2nd), phosphatidylcholine (PC, ranked 33rd), and N,N-diisopropyl-2-benzothiazolesulfenamide (DBTS, ranked 50th) (Figure 2c,d). Their interaction profiles were analyzed through thermodynamic error distribution, and prediction reliability was visualized via correlation heatmaps. Heatmaps of Predicted Alignment Error (PAE), Predicted Distance Error (PDE), scored residues, and aligned residues serve as key tools for interpreting ligand–ligand interaction modes and strengths [36]. At the 1:1 molar ratio (Figure 2c), distinct patterns were observed among the three excipients: Hp-β-CD exhibited uniformly low PAE and PDE values (indicated by dark purple regions), consistent with high spatial consistency and conformational matching, attributable to its rigid β-barrel structure and well-defined host–guest inclusion mechanism. PC showed moderately higher distance errors and local PAE fluctuations, reflecting conformational variability inherent in its amphiphilic structure and multi-molecular interaction mode. DBTS displayed markedly high PAE and PDE values, indicating poor complementarity and low binding stability with d-borneol, consistent with its low ranking.

At the 1:5 molar ratio (Figure 2d), significant ranking changes occurred: Hp-β-CD fell to 23rd place, while PC rose to 6th. Overall, ipTM, ranking_score, PAE, and PDE values were generally reduced compared to the 1:1 predictions. The decline of Hp-β-CD is attributed to saturation and self-aggregation at higher concentrations (Figure 2b), resulting in cavity competition and increased free drug fraction [37,38]. In contrast, PC benefited from increased molecular density, enhancing hydrophobic exposure and facilitating d-borneol incorporation into self-assembled bilayers, thereby improving ipTM scores. The overall reduction in predictive scores at higher ligand densities may stem from increased conformational space and attenuated attention signal across atomic interactions, leading to elevated local errors.

### 3.2. Correlation Validation Between Predicted Interaction Scores and Calculated Binding Affinity

To validate the accuracy of Protenix in predicting small molecule–small molecule interactions—a novel application of the model—we used Autodock Vina [39] as a benchmark for comparison. The binding affinity between each of the top 50 excipients (previously screened by Protenix at a 1:1 molar ratio using ipTM and ranking_score) and d-borneol was calculated. Spearman’s correlation analysis was then performed to assess the relationship between the Protenix-derived indices (ipTM and ranking_score) and the binding affinities.

As shown in (Figure 2e), a strong negative correlation was observed between ipTM and binding affinity (r = −0.8903). Since more negative binding affinity values indicate stronger molecular interactions, and higher ipTM values reflect greater prediction confidence, this negative correlation confirms that ipTM reliably reflects interaction strength. Similarly, ranking_score was also significantly negatively correlated with binding affinity (r = −0.8704) (Figure 2f). As the ranking_score represents an integrated metric for interaction strength, this result further supports the consistency between Protenix predictions and molecular docking-based energy calculations. Together, these findings verify that Protenix can accurately rank and predict the strength of small molecule–small molecule interactions.

### 3.3. Experimental Confirmation of Enhanced Stability of d-Borneol–Excipient Complexes

**Figure 3:**
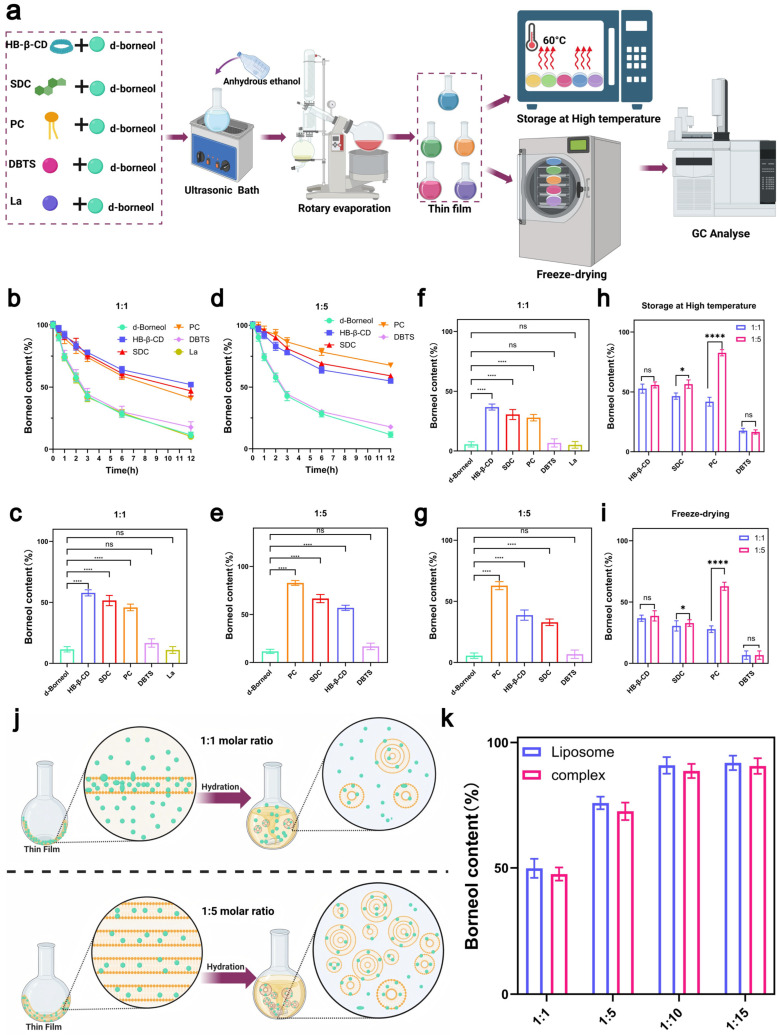
Preparation and stability evaluation of d-borneol–excipient complexes. (**a**) Flowchart of complex preparation and stability assessment. (**b**) Variation in d-borneol content over time for complexes at a 1:1 molar ratio, heated at 60 °C. (**c**) Content comparison and statistical significance of complexes at a 1:1 molar ratio after heating at 60 °C. (**d**) Variation in d-borneol content over time for complexes at a 1:5 molar ratio, heated at 60 °C. (**e**) Content comparison and statistical significance of complexes at a 1:5 molar ratio after heating at 60 °C. (**f**) Content and significance analysis of complexes at a 1:1 molar ratio after freeze-drying. (**g**) Content and significance analysis of complexes at a 1:5 molar ratio after freeze-drying. (**h**) Comparison of d-borneol retention rates between 1:1 and 1:5 complexes after heating at 60 °C for 12 h. (**i**) Comparison of d-borneol retention rates between 1:1 and 1:5 complexes after freeze-drying. (**j**) Proposed mechanism of liposome formation during hydration of d-borneol–phospholipid films at 1:1 and 1:5 molar ratios. (**k**) Comparison of d-borneol content in phospholipid complexes and liposomes at different molar ratios after freeze-drying. * *p* < 0.05, **** *p* < 0.0001, ns not significant.

To further validate the prediction accuracy of Protenix, five representative excipients spanning a range of predicted rankings were selected based on the propensity of volatile compounds to evaporate under insufficient intermolecular stabilization [40]. These included: hydroxypropyl-β-cyclodextrin (HP-β-CD, rank2), sodium deoxycholate (SDC, rank 16), soybean phospholipid (PC, rank 33), N,N-diisopropyl-2-benzothiazolesulfenamide (DBTS, rank 50), and levulinic acid (La, rank 471). Complexes of d-borneol with each excipient were prepared at 1:1 and 1:5 molar ratios. Thermal acceleration tests (60 °C, 12 h) were used to simulate accelerated volatilization, while freeze-drying retention studies were conducted to assess the extent of drug encapsulation (Figure 3a). Together, these experiments quantitatively evaluated the ability of each excipient to enhance the physical stability of d-borneol.

In thermal acceleration tests at a 1:1 molar ratio, the d-borneol retention rate (Figure 3b) decreased over time in a manner consistent with the Protenix ranking: the HP-β-CD group retained >50% of d-borneol after 12 h, owing to strong interactions; the SDC and PC groups showed slower release, while the DBTS and La groups exhibited rapid volatilization, approaching the behavior of pure d-borneol. Statistical analysis of retention rates after 12 h (Figure 3c) confirmed that HP-β-CD, SDC, and PC all significantly outperformed pure d-borneol, with differences aligning with predicted rankings. In contrast, DBTS and La showed no significant improvement over the pure drug, consistent with their low predicted affinity.

At a 1:5 molar ratio, the retention rate of PC increased markedly from 45.89% to 82.83%, exceeding that of SDC (60.57%) (Figure 3d). This improvement is attributed to the concentration-dependent formation of bilayers: at higher concentrations, PC self-assembles into a lipid bilayer that enhances d-borneol encapsulation (Figure 3j). In contrast, retention rates for HP-β-CD (56.89%) and SDC remained largely unchanged from the 1:1 ratio (Figure 3e), suggesting binding saturation had already been achieved—a finding consistent with Protenix predictions. DBTS and La again showed the lowest retention (<20%), supporting the model’s accuracy even in weak-interaction regimes.

Freeze-drying retention results further corroborated these trends. At a 1:1 ratio, retention decreased in the order: HP-β-CD > SDC > PC > DBTS > La (Figure 3f). At a 1:5 ratio, PC retention rose significantly to 62.89% (*p* < 0.0001 vs. 1:1), while those of HP-β-CD and SDC remained largely unchanged, with no significant difference from the 1:1 ratio (Figure 3g). At the 1:1 molar ratio, highly ranked excipients such as HP-β-CD enhanced the stability of d-borneol through strong intrinsic interactions. In contrast, lower-ranked excipients like PC exhibited markedly improved stability at the 1:5 molar ratio, likely due to a synergistic mechanism involving self-assembly behavior at high concentration and intrinsic molecular interactions (Figure 3j). Furthermore, the interaction ranking predicted by Protenix strongly correlated with the actual stability of d-borneol under both thermal (Figure 3h) and freeze-drying (Figure 3i) conditions, confirming the model’s reliability in predicting small molecule–small molecule interactions. Based on these results, PC was selected as the optimal excipient.

### 3.4. Comparison of Stability Differences Between d-Borneol–Phospholipid Complexes and Liposomes

While phospholipid-based excipients can enhance the stability of d-borneol through molecular interactions, phospholipid complexes and liposomes differ fundamentally in their administration feasibility. Although phospholipid complexes improve drug stability, they lack critical attributes required for intravenous injection—such as controllable particle size and optimal dispersibility—and are therefore unsuitable as injectable formulations [41]. Furthermore, experimental results indicated that the stabilizing effect of soybean phospholipid (PC) on d-borneol was significantly enhanced at a 1:5 molar ratio. This suggests that as the phospholipid proportion increases, its role may extend beyond simple molecular interactions, potentially involving multi-molecular assembly into more structured lipid bilayers, thereby improving encapsulation and stabilization. Building on this, we further hypothesized that if guided to complete self-assembly in an aqueous environment, a structurally intact and tightly encapsulated liposome could be formed. Such a system would combine the molecular affinity between the drug and phospholipids with a dual stabilization mechanism, offering superior volatility suppression. Therefore, this study focused on constructing a liposomal delivery system, aiming to achieve long-term stabilization of highly volatile drugs through this synergistic reinforcement strategy [42]. They can facilitate the transport of d-borneol across biological barriers and improve targeted delivery.

To evaluate differences in stabilization efficacy between the two systems, a comparative study was conducted. d-Borneol–phospholipid complexes and liposomes were prepared at molar ratios of 1:1, 1:5, 1:10, and 1:15. The d-borneol content was measured after freeze-drying to assess drug retention and elucidate the stabilization mechanism (Figure 3k). At a low molar ratio (e.g., 1:1), insufficient phospholipid content resulted in limited bilayer loading capacity, leading to poor encapsulation and substantial drug loss (Figure 3f). Consequently, both complexes and liposomes exhibited low d-borneol retention. As the drug–lipid ratio increased, phospholipids assembled into more organized and multilayered structures [43], providing additional encapsulation sites and improving drug retention—as seen in the 1:5, 1:10, and 1:15 groups.

Notably, the d-borneol content did not differ significantly between the 1:10 and 1:15 groups, suggesting that encapsulation was near saturation. Beyond this point, increasing the phospholipid ratio did not enhance drug loading. The retention trends were highly consistent between complexes and liposomes across all ratios, indicating a continuous encapsulation mechanism mediated by the phospholipid bilayer. At higher molar ratios, the well-structured membrane and strengthened hydrophobic and van der Waals interactions between d-borneol and phospholipids promoted more efficient drug anchoring and encapsulation, resulting in a stable drug-loaded system with significantly improved stability.

### 3.5. Molecular Mechanism of Phospholipid Dosage Regulating d-Borneol Liposome Encapsulation and Morphological Characterization

**Figure 4:**
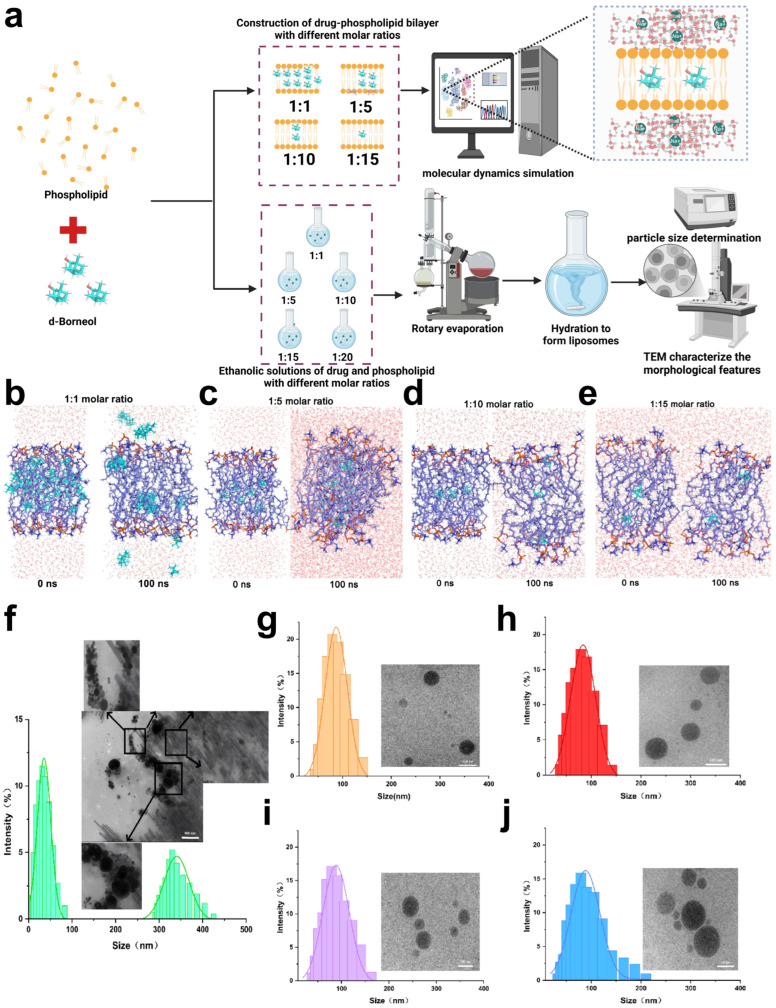
Molecular dynamics (MD) simulation and experimental characterization of d-borneol–phospholipid complexes at varying molar ratios. (**a**) Schematic overview of the MD simulation and experimental design. (**b**) MD simulation snapshots of the d-borneol–phospholipid bilayer at a 1:1 molar ratio at 0 ns and 100 ns. (**c**) MD simulation snapshots of the d-borneol–phospholipid bilayer at a 1:5 molar ratio at 0 ns and 100 ns. (**d**) MD simulation snapshots of the d-borneol–phospholipid bilayer at a 1:10 molar ratio at 0 ns and 100 ns. (**e**) MD simulation snapshots of the d-borneol–phospholipid bilayer at a 1:15 molar ratio at 0 ns and 100 ns. (**f**–**j**) TEM images and size distributions of d-borneol–phospholipid liposomes at molar ratios of 1:1 (**f**), 1:5 (**g**), 1:10 (**h**), 1:15 (**i**), and 1:20 (**j**). The scale bars represent 500 nm (**f**) and 100 nm (**g**–**j**).

The drug-to-lipid (D/L) ratio is a critical parameter for evaluating the drug-loading capacity of liposomes. When the initial D/L ratio is excessively high (>0.95), the surplus drug exceeds the loading capacity of the liposomes, resulting in reduced loading efficiency [44]. This overloading can disrupt the liposomal membrane structure, leading to drug leakage and subsequently compromising both encapsulation efficiency and therapeutic efficacy (Figure 4a). Molecular dynamics simulations (Figure 4b–e) revealed distinct behaviors of d-borneol liposomes at different molar ratios. In the 1:1 system (Figure 4b), d-borneol could not be stably encapsulated within the lipid bilayer over the 0–100 ns simulation period, with substantial drug escape observed after 100 ns. This indicates insufficient bilayer capacity at low phospholipid levels to effectively retain the drug. In contrast, in the 1:5, 1:10, and 1:15 systems (Figure 4c–e), d-borneol remained stably incorporated within the bilayer after 100 ns. These results demonstrate that higher phospholipid content provides sufficient and stable encapsulation sites for d-borneol through multi-molecular interactions such as hydrophobic effects and van der Waals forces, highlighting the essential role of phospholipid dosage in drug encapsulation stability.

Transmission electron microscopy (TEM) images (Figure 4f–j) provided visual confirmation of liposomal morphology. At the 1:1 molar ratio (Figure 4f), d-borneol crystals were visible, and the liposomes exhibited irregular sizes and shapes. This suggests that inadequate phospholipid amounts lead to defective bilayer assembly, resulting in both drug crystallization and irregular nanoparticle formation. Conversely, formulations with 1:5, 1:10, 1:15, and 1:20 ratios all displayed typical liposomal structures with uniform particle sizes (Figure 4g–j), indicating that sufficient phospholipid promotes orderly bilayer self-assembly and stable nano-drug loading systems. Together, molecular dynamics and TEM results establish that phospholipid dosage is a fundamental factor governing the encapsulation efficiency and stability of d-borneol liposomes. Insufficient phospholipid (e.g., 1:1 ratio) causes drug leakage and irregular morphology, compromising stability. In contrast, appropriate phospholipid levels (e.g., 1:5 and above) enable effective drug encapsulation and uniform liposome formation. These findings provide both mechanistic insight and visual evidence for optimizing the D/L ratio in subsequent formulation development.

### 3.6. Molecular Interaction Mechanism of d-Borneol-Liposomes Analyzed by DSC and FTIR

**Figure 5:**
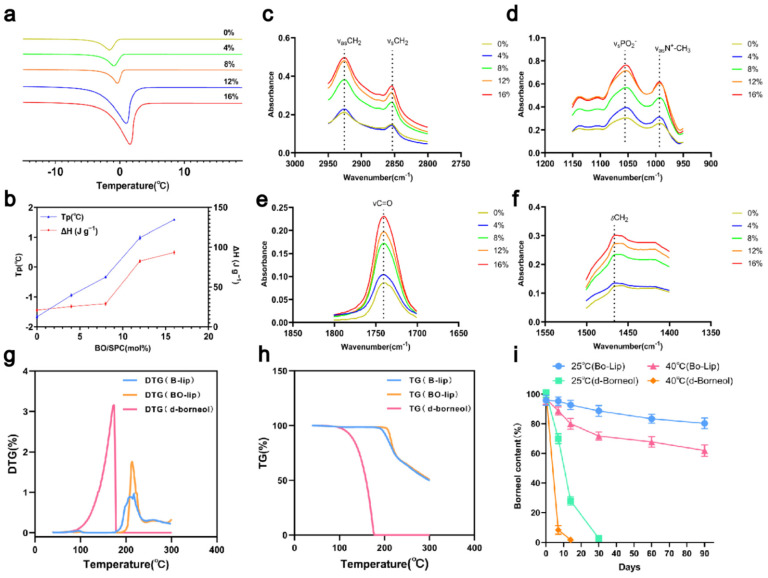
Thermodynamic properties and stability characterization of d-borneol liposomes at different molar ratios (**a**) Differential Scanning Calorimetry (DSC) curves of d-borneol liposomes (Bo-Lip) at different molar ratios. (**b**) Peak temperature (T_p_) and enthalpy change (ΔH) for d-borneol and phospholipid (PC) at various molar ratios. (**c**–**f**) FTIR spectra of d-borneol liposomes: (**c**) CH_3_ stretching (2950–2960 cm^−1^), (**d**) CH_2_ symmetric stretching (2850–2853 cm^−1^), (**e**) C=O stretching (1730–1732 cm^−1^), (**f**) PO_4_^2−^ and N^+^CH_3_ absorption (1000–1200 cm^−1^). (**g**) TG curves of pure d-borneol and liposomes heated from 30 °C to 600 °C at 10 °C/min. (**h**) Derivative thermogravimetry (DTG) curves for corresponding samples. (**i**) Retention of d-borneol in liposomes and pure form during storage at 25 °C and 40 °C (mean ± SD, *n* = 3).

The interaction between drugs and liposomes is closely linked to their physicochemical properties: hydrophilic drugs associate with the polar head groups, amphiphilic drugs partially embed within the lipid bilayer, and lipophilic drugs bind to the hydrophobic tails [45]. (Figure 5a) displays the DSC curves of d-borneol-loaded liposomes (Bo-Lip) at molar ratios of 0%, 4%, 8%, 12%, and 16% (scanning rate: 1 °C/min). Pure PC liposomes exhibited a distinct phase transition peak at −1.629 °C. As the d-borneol concentration increased, the main phase transition peak shifted to higher temperatures, reaching 1.568 °C at the 16% molar ratio. The peak height and sharpness also gradually increased, and the phase transition enthalpy rose from 22.34 J/g to 140.66 J/g (Figure 5b). These results indicate that d-borneol incorporates into the hydrophobic region of the phospholipids and enhances the membrane order and thermal stability by strengthening interactions with the acyl chains in a concentration-dependent manner.

Fourier transform infrared (FTIR) spectroscopy was used to probe intermolecular interactions by examining vibrational modes of phospholipid hydrophobic tails (e.g., -CH_2_-, -CH_3_) and polar head groups (e.g., PO_2_^−^, N^+^-CH_3_). The FTIR findings were consistent with the DSC results (Figure 5c–f). In the CH_2_ stretching region (2800–3000 cm^−1^), as the d-borneol concentration increased, the absorbance of both asymmetric (~2920 cm^−1^) and symmetric (~2850 cm^−1^) stretching vibrations increased, with peaks becoming sharper. This reflects enhanced lipid chain ordering and restricted motion. In the polar head group region (PO_2_^−^ ~1050 cm^−1^, N^+^-CH_3_ ~1000 cm^−1^), the minimal changes in absorbance indicated that d-borneol does not significantly perturb the headgroup chemistry and primarily interacts with hydrophobic chains. Changes in the C=O stretching (~1750 cm^−1^) and CH_2_ bending vibration (~1450 cm^−1^) regions further confirmed increased lipid chain regularity. Together, these results demonstrate that d-borneol embeds within the hydrophobic region of the phospholipid bilayer, modulates lipid chain fluidity, and significantly enhances membrane order and stability with increasing concentration.

### 3.7. Thermogravimetric Analysis and Long-Term Stability Study

TGA-DTG analysis revealed that the volatility of d-borneol is temperature-dependent (Figure 5g,h). Little volatilization occurred between 30 and 86 °C (DTG < 0.1%). A rapid volatilization phase was observed from 87 to 165 °C, with the maximum rate occurring between 129 and 164.5 °C. Volatilization was complete at 170.44 °C (Figure 5g,h). In contrast, d-borneol liposomes (Bo-Lip) exhibited excellent thermal stability. No significant mass loss (DTG < 0.1%) (Figure 5h) was detected between 30 and 192 °C. The mass loss occurring between 192 and 220 °C was attributed to thermal decomposition of sucrose, a cryoprotectant used during freeze-drying [46], as confirmed by the identical TGA profile of blank liposomes (B-Lip). Phospholipid decomposition occurred around 320 °C [47], indicating that no d-borneol volatilization occurred in this temperature range. Long-term stability data (Figure 5i) further demonstrated that free d-borneol was almost completely lost within 30 days under 25 °C storage conditions, whereas Bo-Lip retained approximately 80% of the drug after 90 days. Under accelerated conditions (40 °C), free d-borneol degraded completely within 30 days, while Bo-Lip still retained over 60% after 90 days. These results indicate that liposomal encapsulation significantly suppresses d-borneol volatilization through bilayer entrapment and drug–phospholipid interactions, thereby providing critical stability support for the formulation development of this drug.

## 4. Conclusions

This study addresses the stability challenges associated with the volatile drug d-borneol, as well as the inefficiency and reliance on trial-and-error methods inherent in traditional excipient screening. For the first time, the Protenix structure prediction model was used to perform high-throughput prediction and ranking of interactions between d-borneol and 472 FDA-approved excipients at 1:1 and 1:5 molar ratios. The goal was to identify excipients with strong interactions and to construct nano-formulations that inhibit d-borneol volatilization. The results revealed that the molar ratio significantly influenced the excipient ranking. At a 1:1 ratio, HP-β-CD ranked 2nd due to host–guest inclusion, whereas phosphatidylcholine (PC) ranked 33rd, which reflected its weak hydrophobic synergy. In contrast, at a 1:5 ratio, excess HP-β-CD self-aggregated and dropped to 23rd due to cavity competition. PC, however, rose to 6th place: increased molecular density promoted hydrophobic interactions with d-borneol, followed by self-assembly into a bilayer that provided additional embedding sites and synergistically enhanced stability. Molecular docking validation showed a significant negative correlation between the predicted indices (iPTM and ranking_score) of the top 50 excipients and their binding affinities. This confirmed that Protenix predictions were consistent with binding energies derived from molecular docking, supporting the model’s accuracy. Four excipients with varying rankings were selected to form complexes at different molar ratios. Thermal acceleration and freeze-drying experiments demonstrated strong agreement between the experimental retention rate of d-borneol and the predicted excipient rankings. Notably, the d-borneol retention rate with PC was substantially higher at the 1:5 ratio than at 1:1, and also exceeded that of other highly ranked excipients—consistent with model predictions.

PC was ultimately chosen as the optimal excipient for constructing d-borneol liposomes. Molecular dynamics simulations confirmed stable embedding of d-borneol within the hydrophobic region of the phospholipids. Transmission electron microscopy revealed intact liposomal structures with uniform size distribution and no drug crystallization. Differential scanning calorimetry and Fourier-transform infrared spectroscopy analyses indicated that d-borneol enhanced lipid membrane order and thermal stability via hydrophobic interactions, without disrupting the polar headgroups. Thermogravimetric analysis and long-term stability tests showed that the liposomal formulation significantly improved stability compared to pure d-borneol: retention exceeded 80% after 90 days at 25 °C and remained above 60% under accelerated conditions (40 °C). By integrating artificial intelligence with nano-formulation technology, this work provides a systematic strategy to overcome the stability limitations of volatile drugs and offers new insights and methodologies for developing related pharmaceutical formulations.

This study confirms the effectiveness of the Protenix model-based AI screening strategy in predicting drug-excipient interactions, while also acknowledging its inherent limitations. The model’s predictions are based on a static binary interaction framework, which may lead to biased assessments when applied to highly flexible molecules or systems dependent on dynamic multi-molecular assembly. Furthermore, complex formulation factors—such as excipient self-aggregation behavior and process-related kinetic effects—extend beyond the current computational scope of the model. Therefore, this approach is best regarded as a supportive tool for preliminary screening and mechanistic investigation, with its predictions requiring integration with formulation expertise and experimental validation for final decision-making.

Despite these constraints, the strategy exhibits promising potential for generalizability in screening compatible excipients for other volatile or hydrophobic active compounds. However, its extension to macromolecular excipients such as polymers, or to more complex formulation objectives like sustained release or targeted delivery, presents new challenges. These include accurate sampling of macromolecular conformations and the integration of multiple performance indicators. These directions also outline meaningful pathways for the further integration of AI into rational formulation design in the future.

## Data Availability

The excipient data used in this study were retrieved from the FDA Excipient Database (Inactive Ingredients Database Download|FDA). The code and data supporting the findings of this study are available in the Supplementary Materials. The original contributions presented in this study are included in the article/Supplementary Material. Further inquiries can be directed to the corresponding authors.

## Supplementary Materials

The following supporting information can be downloaded at https://www.mdpi.com/article/10.3390/pharmaceutics18010111/s1. Table S1: Protenix_prediction_results_1_1.xlsx (Top 50 excipients ranked by Protenix model at 1:1 molar ratio); Table S2: Protenix_prediction_results_1_5.xlsx (Top 50 excipients ranked by Protenix model at 1:5 molar ratio); Code S1: remove_duplicates.py (Remove duplicate UNII entries from ingredient database); Code S2: merge_data.py (Merge ingredient data with SMILES records via UNII); Code S3: remove_rows_without_smiles.py (Filter entries lacking SMILES data); Code S4: generate_input_json.py (Generate protein-ligand input JSON files from UNII-SMILES data); Code S5: generate_input_json_v2.py (Create optimized input JSON for molecular docking); Code S6: batch_processing_output_json.py (Process JSON output files from protein-ligand docking); Code S7: batch_processing_output_json_v2.py (Compute average binding metrics from docking results); Code S8: add_name_to_output_xlsx.py (Map ingredient names to output Excel via UNII); Code S9: add_name_to_output_xlsx_v2.py (Enhanced UNII suffix removal for ingredient naming).

## References

[1] Turek C., Stintzing F.C. (2013). Stability of Essential Oils: A Review. Compr. Rev. Food Sci. Food Saf..

[2] Boga M., Ersoy E., Eroglu Ozkan E., Cinar E., Mataraci Kara E., Yesil Canturk Y., Zengin G. (2021). Volatile and phenolic profiling of a traditional medicinal plant, *Hypericum empetrifolium* with in vitro biological activities. J. Ethnopharmacol..

[3] Barreto I.C., de Almeida A.S., Sena Filho J.G. (2021). Taxonomic Insights and Its Type Cyclization Correlation of Volatile Sesquiterpenes in Vitex Species and Potential Source Insecticidal Compounds: A Review. Molecules.

[4] Bhalani D.V., Nutan B., Kumar A., Singh Chandel A.K. (2022). Bioavailability Enhancement Techniques for Poorly Aqueous Soluble Drugs and Therapeutics. Biomedicines.

[5] Zhao J.J., Yang J., Xie Y. (2019). Improvement strategies for the oral bioavailability of poorly water-soluble flavonoids: An overview. Int. J. Pharm..

[6] Xiao Z., Zhang Y., Niu Y., Ke Q., Kou X. (2021). Cyclodextrins as carriers for volatile aroma compounds: A review. Carbohydr. Polym..

[7] Paiva-Santos A.C., Ferreira L., Peixoto D., Silva F., Soares M.J., Zeinali M., Zafar H., Mascarenhas-Melo F., Raza F., Mazzola P.G. (2022). Cyclodextrins as an encapsulation molecular strategy for volatile organic compounds—Pharmaceutical applications. Colloids Surf. B Biointerfaces.

[8] Gharib R., Auezova L., Charcosset C., Greige-Gerges H. (2017). Drug-in-cyclodextrin-in-liposomes as a carrier system for volatile essential oil components: Application to anethole. Food Chem..

[9] Puri S., Mazza M., Roy G., England R.M., Zhou L., Nourian S., Anand Subramony J. (2023). Evolution of nanomedicine formulations for targeted delivery and controlled release. Adv. Drug Deliv. Rev..

[10] Han Y., Huang S. (2023). Nanomedicine is more than a supporting role in rheumatoid arthritis therapy. J. Control. Release.

[11] Hassanzadeh H., Alizadeh M., Hassanzadeh R., Ghanbarzadeh B. (2022). Garlic essential oil-based nanoemulsion carrier: Release and stability kinetics of volatile components. Food Sci. Nutr..

[12] Emtiazi H., Salari Sharif A., Hemati M., Fatemeh Haghiralsadat B., Pardakhti A. (2022). Comparative Study of Nano-liposome and Nano-niosome for Delivery of *Achillea millefolium* Essential Oils: Development, Optimization, Characterization and Their Cytotoxicity Effects on Cancer Cell Lines and Antibacterial Activity. Chem. Biodivers..

[13] Hu G., Zhou Z., Tang G., Liu Y., Zhang X., Huang Y., Yan G., Xiao J., Yan W., Li J. (2025). Prodrug Self-Assemblies Based on Plant Volatile Aldehydes with Improved Stability and Antimicrobial Activity Against Plant Pathogens. Small.

[14] Tran P.H.L., Wang T., Yin W., Tran T.T.D., Barua H.T., Zhang Y., Midge S.B., Nguyen T.N.G., Lee B.-J., Duan W. (2019). Development of a nanoamorphous exosomal delivery system as an effective biological platform for improved encapsulation of hydrophobic drugs. Int. J. Pharm..

[15] Weisany W., Yousefi S., Tahir N.A.-r., Golestanehzadeh N., McClements D.J., Adhikari B., Ghasemlou M. (2022). Targeted delivery and controlled released of essential oils using nanoencapsulation: A review. Adv. Colloid Interface Sci..

[16] Beraldo-de-Araújo V.L., Beraldo-de-Araújo A., Costa J.S.R., Pelegrine A.C.M., Ribeiro L.N.M., de Paula E., Oliveira-Nascimento L. (2019). Excipient-excipient interactions in the development of nanocarriers: An innovative statistical approach for formulation decisions. Sci. Rep..

[17] Chowdhury P., Nagesh P.K.B., Hatami E., Wagh S., Dan N., Tripathi M.K., Khan S., Hafeez B.B., Meibohm B., Chauhan S.C. (2019). Tannic acid-inspired paclitaxel nanoparticles for enhanced anticancer effects in breast cancer cells. J. Colloid Interface Sci..

[18] Nel A.E. (2020). Transformational Impact of Nanomedicine: Reconciling Outcome with Promise. Nano Lett..

[19] Zhu Y., Huang R., Zhu R., Xu W., Zhu R., Cheng L. (2018). DeepScreen: An Accurate, Rapid, and Anti-Interference Screening Approach for Nanoformulated Medication by Deep Learning. Adv. Sci..

[20] Mann J.L., Maikawa C.L., Smith A.A.A., Grosskopf A.K., Baker S.W., Roth G.A., Meis C.M., Gale E.C., Liong C.S., Correa S. (2020). An ultrafast insulin formulation enabled by high-throughput screening of engineered polymeric excipients. Sci. Transl. Med..

[21] Liu M., Yang Z., Wen J., Ma Z., Sun L., Wang M., Ren X. (2024). The effect of honey as an excipient in the processing of traditional chinese medicine based on chemical profiling, artificial neural network, and virtual screening: Cortex Mori as an example. Arab. J. Chem..

[22] Batra R., Chan H., Kamath G., Ramprasad R., Cherukara M.J., Sankaranarayanan S.K.R.S. (2020). Screening of Therapeutic Agents for COVID-19 Using Machine Learning and Ensemble Docking Studies. J. Phys. Chem. Lett..

[23] Abramson J., Adler J., Dunger J., Evans R., Green T., Pritzel A., Ronneberger O., Willmore L., Ballard A.J., Bambrick J. (2024). Accurate structure prediction of biomolecular interactions with AlphaFold 3. Nature.

[24] Eshak F., Goupil-Lamy A. (2025). Advancements in Nanobody Epitope Prediction: A Comparative Study of AlphaFold2Multimer vs AlphaFold3. J. Chem. Inf. Model..

[25] Jabeen A., Oakeshott J.G., Lee S.F., Ranganathan S., Taylor P.W. (2024). Template-based modeling of insect odorant receptors outperforms AlphaFold3 for ligand binding predictions. Sci. Rep..

[26] Chen X., Zhang Y., Lu C., Ma W., Guan J., Gong C., Yang J., Zhang H., Zhang K., ByteDance AML AI4Science Team (2025). Protenix-Advancing Structure Prediction Through a Comprehensive AlphaFold3 Reproduction. bioRxiv.

[27] Kulkarni M., Sawant N., Kolapkar A., Huprikar A., Desai N. (2021). Borneol: A Promising Monoterpenoid in Enhancing Drug Delivery Across Various Physiological Barriers. AAPS PharmSciTech.

[28] Wen Y., Zhang Z., Cai Z., Liu B., Wu Z., Liu Y. (2022). Ligustrazine-Loaded Borneol Liposome Alleviates Cerebral Ischemia–Reperfusion Injury in Rats. ACS Biomater. Sci. Eng..

[29] Long Y., Liu S., Wan J., Zhang Y., Li D., Yu S., Shi A., Li N., He F. (2023). Brain targeted borneol-baicalin liposome improves blood-brain barrier integrity after cerebral ischemia-reperfusion injury via inhibiting HIF-1α/VEGF/eNOS/NO signal pathway. Biomed. Pharmacother..

[30] Chen Y., Jin X., Kuang Y., Zhang S., Zhang C., Li C., Guo B. (2023). A Novel Oral Drugs Delivery System for Borneol Based on HiCap^®^100 and Maltodextrin: Preparation, Characterization, and the Investigation as an Intestinal Absorption Enhancer. AAPS PharmSciTech.

[31] Wang W., Ren Z., Wang L., Cai Y., Ma H., Fang L., Su J. (2021). Nanoparticles-stabilized encapsulation of borneol and citral: Physicochemical characteristics, storage stability and enhanced antibacterial activities. J. Food Sci..

[32] Chen J., He J., Li N., Zheng H., Zhao S. (2019). Determination and Correlation of Solubility of Borneol, Camphor, and Isoborneol in Different Solvents. J. Chem. Eng. Data.

[33] Ma R., Lu D., Wang J., Xie Q., Guo J. (2023). Comparison of pharmacological activity and safety of different stereochemical configurations of borneol: L-borneol, D-borneol, and synthetic borneol. Biomed. Pharmacother..

[34] Homma F., Huang J., van der Hoorn R.A.L. (2023). AlphaFold-Multimer predicts cross-kingdom interactions at the plant-pathogen interface. Nat. Commun..

[35] Wallner B. (2023). AFsample: Improving multimer prediction with AlphaFold using massive sampling. Bioinformatics.

[36] He X.-H., Li J.-R., Shen S.-Y., Xu H.E. (2025). AlphaFold3 versus experimental structures: Assessment of the accuracy in ligand-bound G protein-coupled receptors. Acta Pharmacol. Sin..

[37] Sahu K.M., Patra S., Swain S.K. (2023). Host-guest drug delivery by β-cyclodextrin assisted polysaccharide vehicles: A review. Int. J. Biol. Macromol..

[38] Fang F., Zhang Z., Zhang P., Zhang X., Ma H., Wei Y. (2024). Fluorescence detection of amantadine based on competitive β-Cyclodextrin host-guest inclusion process. Colloids Surf. A Physicochem. Eng. Asp..

[39] Eberhardt J., Santos-Martins D., Tillack A.F., Forli S. (2021). AutoDock Vina 1.2.0: New Docking Methods, Expanded Force Field, and Python Bindings. J. Chem. Inf. Model..

[40] Li Z., Huang J., Ye L., Lv Y., Zhou Z., Shen Y., He Y., Jiang L. (2020). Encapsulation of Highly Volatile Fragrances in Y Zeolites for Sustained Release: Experimental and Theoretical Studies. ACS Omega.

[41] Gore D.D., Mishra N., Kumar D., Jena G., Jachak S.M., Tikoo K., Bansal A.K., Singh I.P. (2025). Anti-inflammatory activity, stability, bioavailability and toxicity studies on seabuckthorn polyphenol enriched fraction and its phospholipid complex (Phytosomes) preparation. Int. J. Biol. Macromol..

[42] Barenholz Y.C. (2012). Doxil^®^—The first FDA-approved nano-drug: Lessons learned. J. Control. Release.

[43] Hagn F., Nasr M.L., Wagner G. (2018). Assembly of phospholipid nanodiscs of controlled size for structural studies of membrane proteins by NMR. Nat. Protoc..

[44] Chountoulesi M., Naziris N., Pippa N., Demetzos C. (2018). The significance of drug-to-lipid ratio to the development of optimized liposomal formulation. J. Liposome Res..

[45] Zhang W., Falconer J.R., Baguley B.C., Shaw J.P., Kanamala M., Xu H., Wang G., Liu J., Wu Z. (2016). Improving drug retention in liposomes by aging with the aid of glucose. Int. J. Pharm..

[46] Eggleston G., Trask-Morrell B.J., Vercellotti J.R. (1996). Use of Differential Scanning Calorimetry and Thermogravimetric Analysis to Characterize the Thermal Degradation of Crystalline Sucrose and Dried Sucrose−Salt Residues. J. Agric. Food Chem..

[47] Naktiyok J., Bayrakçeken H., Özer A.K., Gülaboğlu M.Ş. (2013). Kinetics of thermal decomposition of phospholipids obtained from phosphate rock. Fuel Process. Technol..

